# A Novel Multilayered Multidisk Oral Tablet for Chronotherapeutic Drug Delivery

**DOI:** 10.1155/2013/569470

**Published:** 2013-08-20

**Authors:** Zaheeda Khan, Yahya E. Choonara, Pradeep Kumar, Lisa C. du Toit, Valence M. K. Ndesendo, Viness Pillay

**Affiliations:** ^1^Department of Pharmacy and Pharmacology, Faculty of Health Sciences, University of the Witwatersrand, 7 York Road, Parktown, Johannesburg 2193, South Africa; ^2^Department of Pharmaceutics and Formulation Sciences, St John's University of Tanzania, Dodoma, Tanzania

## Abstract

A Multilayered Multidisk Tablet (MLMDT) comprising two drug-loaded disks enveloped by three drug-free barrier layers was developed for use in chronotherapeutic disorders, employing two model drugs, theophylline and diltiazem HCl. The MLMDT was designed to achieve two pulses of drug release separated by a lag phase. The polymer disk comprised hydroxyethylcellulose (HEC) and ethylcellulose (EC) granulated using an aqueous dispersion of EC. The polymeric barrier layers constituted a combination of pectin/Avicel (PBL) (1st barrier layer) and hydroxypropylmethylcellulose (HPMC) (HBL1 and HBL2) as the 2nd and 3rd barrier layers, respectively. Sodium bicarbonate was incorporated into the diltiazem-containing formulation for delayed drug release. Erosion and swelling studies confirmed the manner in which the drug was released with theophylline formulations exhibiting a maximum swelling of 97% and diltiazem containing formulations with a maximum swelling of 119%. FTIR spectra displayed no interactions between drugs and polymers. Molecular mechanics simulations were undertaken to predict the possible orientation of the polymer morphologies most likely affecting the MLMDT performance. The MLMDT provided two pulses of drug release, separated by a lag phase, and additionally it displayed desirable friability, hardness, and uniformity of mass indicating a stable formulation that may be a desirable candidate for chronotherapeutic drug delivery.

## 1. Introduction

Biological processes, namely heart rate, fibrinolytic activity, platelet aggregability, airway resistance, gastric secretion, and gastric motility, are known to follow the timed daily scale [1–5]. In addition symptoms of diseases such as hypertension, coronary heart disease, asthma, arthritis, osteoarthritis, and duodenal ulcers also fluctuate with the time of the day [6–20]. These diseases, although usually treated with sustained release preparations, would benefit from pulsatile drug delivery where drug is released at predetermined time intervals after a lag phase. Pulsatile drug delivery offers the following advantages: (i) extended daytime or nighttime activity, (ii) reduced side effects, (iii) reduced dosing frequency, (iv) reduction in dose size, (v) improved patient compliance, and (vi) lower medication costs due to the reduced dosing. In addition to the use in these disorders, pulsatile drug delivery may be beneficial for drugs with an extensive first pass metabolism as well as to target drugs to a specific site within the gastrointestinal tract [21–24].

Numerous research works have been published on pulsatile drug delivery [22, 25–30]. Among these is a “tablet in capsule” device used to provide a three-pulse drug release [31]. The capsule consists of an impermeable capsule body and a soluble cap. The capsule is loaded with a three-layered tablet which serves to provide the first two pulses and a double-layered tablet which serves to provide the third pulse. Additional pulsatile drug delivery systems intended for chronotherapy include capsules [14, 21, 32], tablets [33–35], and low density carriers [16, 36]. Marketed chronotherapeutic drug delivery systems include OROS (Alza Corporation, Mountain View, CA, USA), CEFORM (Biovail Corporation, Mississauga, ON, Canada), CODAS (Elan Corporation, Gainesville, FL, USA), Egalet (Egalet a/s, Vaerlose, Copenhagen, Denmark), CONTIN (Purdue Pharma, Pickering, ON, Canada), Pulsincaps (R.P. Scherer Corporation, Troy, MI, USA), Diffucaps (Eurand Pharmaceuticals, Yardley, PA, USA), and TIMERx (Penwest Pharmaceutical Company, Danbury, CT, USA).

The characterization of the biological rhythms is usually based on (1) the periods related to the completion of one cycle, (2) the levels conferring to the rhythmic variation baseline, (3) the amplitudes depicting the extent of variability, and (4) the phases involving peaks and troughs corresponding to the respective time scale [5, 37]. The circadian rhythms can further be differentiated based on small amplitudes (e.g., heart rate) or high amplitudes (e.g., blood cortisol concentrations) [5, 38]. The above mentioned characteristics may be used as a reference towards the determination of the influence of “circadian rhythm” on the physiological systems and the physiology of diseased states [5]. 

The novel system proposed is referred to as a Multilayered Multidisk Tablet (MLMDT). The device comprises two drug-loaded disks that serve as the two pulsatile doses. These disks were enveloped between three polymeric barrier layers. The first barrier layer (upper) erodes exposing a disk which results in an immediate pulse of drug release. The second and third barrier layers (middle and bottom) swell and protect the remaining disk from releasing drug producing a lag as well as a controlled drug release phase. Erosion of these barrier layers triggers the second pulse of release. For example, if the patient has to self-administer the MLMDT after dinner, the first dose will provide immediate release and will persist until the patient retires to bed. Drug release will then “turn off” whilst the patient is asleep and then “turn on” before awakening when the controlled release disk is activated. This coincides with the body's natural circadian rhythm and delivers drug when it is mainly needed.

The purpose of this study therefore was to explore the design and development of the MLMDT for use in chronotherapeutic disorders. Theophylline (THP) and diltiazem hydrochloride (DTZ) were employed as the model drugs to develop two separate formulations, showing the versatility of the MLMDT when incorporated with prototype water insoluble and water soluble drugs. In addition to the experimental studies, the incorporation of EC along with HEC, pectin and Avicel was mechanistically elucidated using mechanistic modeling of the three-dimensional architecture of the respective saccharide molecular complexes for the prediction of the relative orientation of the polymer morphologies affecting the MLMDT performance.

## 2. Materials and Methods

### 2.1. Materials

Materials employed include ethylcellulose (EC) (48–49.5 ethoxy content, viscosity = 10 cP), theophylline (THP), and diltiazem hydrochloride (DTZ) that were purchased from Sigma Aldrich (Sigma Aldrich, MO, USA) as well as hydroxypropyl methylcellulose (HPMC) (methocel K4M CR, surelease), ethylcellulose (EC aqueous dispersion) (sureteric) (Colorcon Limited, Kent, United Kingdom); hydroxyethylcellulose (HEC) (degree of molar substitution = 2.5) (Merck, Darmstadt, Germany), and pectin classic CU701 (degree of esterification 38%, Mw = 80 g/mol) (Herbstreith and Fox GmbH, Werder/Havel, Germany). Lactose and sodium bicarbonate (Saarchem, Krugersdorp, South Africa) as well as microcrystalline cellulose (Avicel 101) (FMC Biopolymer, Drammen, Norway) were used as excipients. All other reagents used were of analytical grade and were employed as purchased.

### 2.2. Methods

#### 2.2.1. Preparation of the Multilayered Multidisk Tablet (MLMDT)

*(1) Preparation of Disk One (Lactose Disk).* Lactose and either THP or DTZ (30% of total drug) were measured, blended, and directly compressed at 5 tons using a Karnavati Mini Press II (Rimek Products, Gujarat, India) loaded with punch and dies with a diameter of 10 mm.

*(2) Preparation of Disk Two (Polymer Disk).* Quantities of drug (70% of total drug) and polymer (either HEC or EC) were granulated using a wet granulation approach with either surelease, sureteric, or a combination as the granulating fluid. Surelease was prepared as per manufacturer instruction by measuring a 60% *w*/*v* of the solution and adding 40% *w*/*v* of deionized water (Milli-Q Millipore, Billerica, MA, USA). The solution was then agitated for 40 minutes prior to use. Sureteric was reconstituted to a 15% solid suspension using deionized water (Milli-Q Millipore, Billerica, MA, USA) to which 0.33% of an antifoaming agent was added. The suspension was agitated for 30 minutes prior to use. Drug and polymer were blended using a cube blender (Erweka Apparatebau, Heusenstamm, Germany) to which the granulating fluid was then added to produce a wet mass. The wet mass was then passed through a 2 *μ*m aperture sieve and collected. The granules were placed in an oven at 37°C until dry. The granules were weighed and compressed in a disk at 5 tons using a Karnavati Mini Press II.

**Figure 1:**
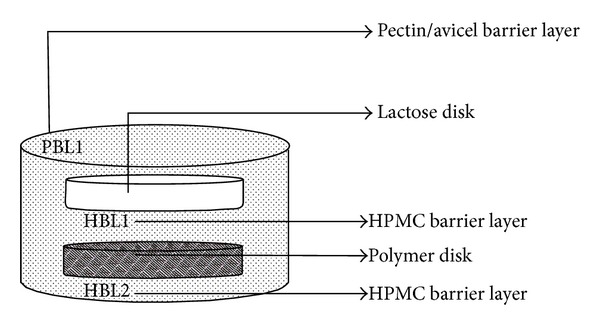
Schematic of the Multilayered Multidisk Tablet (MLMDT) consisting of two drug disks surrounded by barrier layers (PBL1, HBL1, and HBL2).

*(3) Preparation of the Multilayered Multidisk Tablet (MLMDT).* A schematic of the MLMDT is shown in Figure 1. Briefly, the barrier layers comprised a pectin/Avicel blend as the first upper barrier layer and HPMC as the middle and bottom barrier layers. DTZ formulations contained sodium bicarbonate combined with HPMC in various regions.  Table 1 expands on the specific quantities utilized.

In order to combine the MLMDT, a barrier layer was added to the 13 mm punch and die, and after leveling out the powder blend, a disk was added and then centered using the tip of a needle. The next barrier layer was added and leveled followed by the placement and centering of a disk and lastly the third barrier layer. The punch was then inserted and the tablet was compressed at 5 tons using a Beckman hydraulic tablet press (Beckman Instruments, Inc., Fullerton, CA, USA), fitted with a flat-faced punch and die (diameter 13 mm). To minimize processing variables, all tablets were produced under identical conditions. The production of these tablets was performed in triplicate.

#### 2.2.2. In-Process Validation Tests on the MLMDT

Friability, hardness, thickness, and uniformity of mass analyses were performed on both disks as well as the MLMDT. A sample of 10 units each was examined to ensure reproducibility of the tablet making process. Analyses were performed on a Hardness Tester (Pharma Test, Hainburg, Germany) while friability was determined on a Friabilator (Erweka D-63150, Heusenstamm, Germany) at 25 rpm for 4 minutes with 1% set as the upper limit of acceptability. The weight of each MLMDT was determined using an analytical digital balance (Mettler, Model AE 240, Griefensee, Switzerland) with readings recorded to 2 decimal places. A digital caliper (25 × 0.01 mm capacity) (Taizhou hangyu tools gauge and blades Co., Ltd., Wenqiao, Zhejiang, China) was used to determine the thickness of both the disks and the MLMDT.

#### 2.2.3. Computational Modeling to Obtain an Optimized Formulation Using an Artificial Neural Network (ANN) Approach

**Figure 2:**
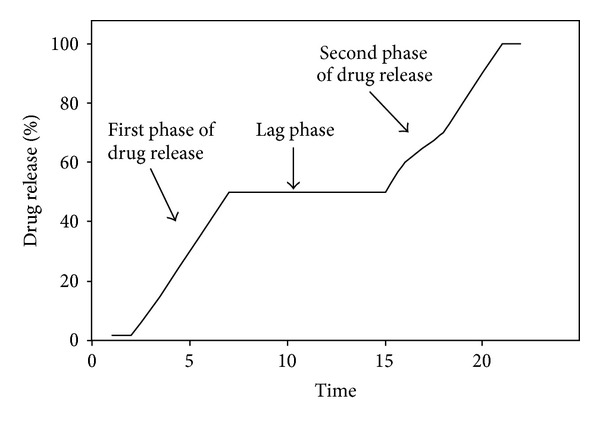
Schematic model depicting a representative ideal drug release profile from the MLMDT.

The major aim of this phase of the study was to obtain the desired drug release profile (i.e., pulsatile drug release) as modeled in Figure 2. By preventing drug release from the polymer disk for the first 12–15 hours, this lag phase may be achieved. Preliminary studies suggested that the duration of the lag phase is predominantly dependent on the composition of the polymer disk with HPMC, EC, and HEC displaying optimum properties. ANN was explored as an optimization option based on its robustness of convergence algorithms for complex formulations that have diverse variables in addition to a heterogeneous archetype. Thus, a more dynamic optimization model was employed to appropriately optimize the MLMDT performance. 

Based on this analogy, 21 formulations with variations in the quantities of HPMC, EC, and HEC were evaluated for drug release.  Table 2(a) depicts 11 THP formulations while Table 2(b) illustrates 10 DTZ formulations. Although HPMC proved to be a suitable candidate when formulating THP devices, this was not the case for DTZ formulations and therefore was omitted. Sodium bicarbonate was evaluated for its effect on drug release and was studied both in the polymer disk and the barrier layers. 

**Figure 3:**
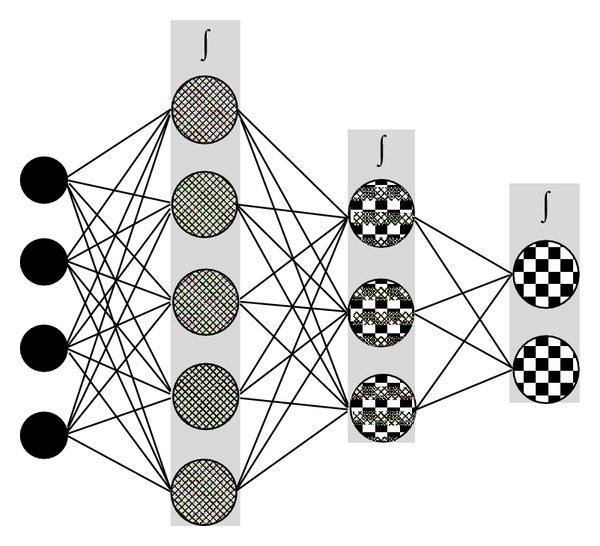
Schematic representing a Multilayer Perceptron (MLP) with two hidden layers.

The data generated from the formulations listed in Table 2 was used to construct a Multilayer Perceptron (MLP) as an ANN approach in order to generate an optimized formulation. MLPs are layered feedforward networks typically trained with static back propagation. These networks have found their way into countless applications requiring static pattern classification. Their main advantage is that they can approximate any input/output map. The key disadvantages are that they train slowly and require lots of training data (typically requiring three times more training samples than network weights). A generalized feedforward (GFF) network is a generalization of the MLP such that connections can jump over one or more layers (Figure 3). In theory, MLP can solve any problem that a generalized feedforward network cannot solve. In practice, however, generalized feedforward networks often solve the problem much more efficiently. 

For the hidden and output layers, a genetic algorithm with the Sigmoid Axon transfer function and Conjugate Gradient learning rule were employed. A maximum of 10 000 epochs were run on NeuroSolutions version 4.32 (NeuroDimension Inc., Gainsville, FL, USA) to ensure optimal training of data. This study utilized a GFF model to predict the drug release.

#### 2.2.4. *In Vitro* Drug Release Analysis

The *in vitro* drug release studies were performed using a USP dissolution apparatus II (Erweka, Heusenstamm, Germany) equipped with paddles. Dissolution was performed in 900 mL simulated human gastrointestinal fluid (SHGF) pH 1.2 [39] for the first 2 hours and simulated human intestinal fluid (SHIF) pH 6.8 [40] for the remainder of the study. Where sodium bicarbonate was used, formulations were tested in pH 1.2 only. The MLMDT was placed on a ring wire mesh assembly [41]. The wire mesh fits into the lower portion of the glass vessels and prevents tablets from sticking to the vessel, allowing the full surface area of the tablet to be exposed to the dissolution medium. Dissolution studies were performed at a paddle speed of 50 rpm and at a temperature of 37 ± 0.5°C. Sampling of 5 mL was undertaken every 1 hour for 12 hours and thereafter at the 24th hour. The withdrawn quantity of sample (i.e., 5 mL) was replaced with an equal amount of the simulated fluid such that the volume of the simulated fluid in the dissolution medium remained constant. The drug content was analyzed by UV spectrophotometer at *λ*_280 nm_ for THP and *λ*_238 nm_ for DTZ and computed from a standard linear curve of drug in SHGF and SHIF (*R* ^2^ > 0.99). Photographs of the tablets at certain dissolution time were captured using a camera (Samsung NV15, Korea) to obtain changes of the dry-coated tablets during dissolution by recording the aerial view of the MLMDT.

#### 2.2.5. Polymer Swelling Studies

The rate of water uptake was determined by the equilibrium weight gain method [42]. Tablets were weighed and placed in a USP dissolution apparatus II in the same way as described in Section 2.2.4 at pH 6.8 over a period of 24 hours. At predetermined regular intervals, the MLMDTs were removed, blotted with absorbent sheets to remove excess fluid, and reweighed. The percentage water uptake, which is the degree of swelling, was estimated at each time point using (1) as follows:

(1) $$\begin{array}{c} \%\;\;\text{water}\;\text{uptake}=\frac{W_{s}-W_{i}}{W_{p}}\times 100, \end{array}$$

where *W*_*s*_ is the weight of the swollen matrix at time *t*, *W*_*i*_ is the initial weight of the matrix, and *W*_*p*_ is the weight of polymer in the matrix. The polymer swelling or water uptake data was obtained by calculating the mean of three determinations since the experiment was performed in triplicate.

#### 2.2.6. Matrix Erosion Studies

Erosion studies were undertaken employing SGHF and SHIF, identical to the dissolution testing study described earlier. MLMDTs were subjected to analysis using USP dissolution apparatus II 2 at 50 rpm. At predetermined time intervals, the MLMDTs were removed and dried to constant weight in a thermofan oven. The percentage matrix erosion (*E*) at time *t* was estimated using (2) as follows:

(2) $$\begin{array}{c} \text{matrix}\;\text{erosion}\;\left(\%\right)=\frac{W_{i}-W_{t}}{W_{i}}\times 100, \end{array}$$

where *W*_*i*_ is the initial starting weight of the matrix and *W*_*t*_ is the weight of matrix subjected to erosion, for time *t*. The matrix erosion data was obtained by calculating the mean of three determinations.

#### 2.2.7. Textural Analysis

Textural analysis was used to evaluate energy of deformation and indentation hardness of the MLMDT which was converted to Brinell Hardness Number (BHN). A calibrated texture analyzer (TA.XT*plus*, Stable Microsystems, Surrey, UK) fitted with a flat-tipped steel probe (2 mm diameter) was employed for energy of matrix deformation and a ball probe (2 mm diameter) for indentation hardness. Hardness was measured as the force (N) required to indent the matrices to a set distance (mm). This force was then converted to BHN using (3). Data was captured at a rate of 200 points per second via texture exponent software (version 3.2). The employed settings are shown in Table 3. All experiments were conducted in triplicate. Consider the following:

(3) $$\begin{array}{c} \text{BHN}=\frac{2F}{\pi D{\left(D^{2}-d^{2}\right)}^{1/2}}, \end{array}$$

where *F* is the force generated from indentation, *D* is the diameter of spherical probe indenter (3.175 mm), and *d* is the indentation depth (1.563 mm).

#### 2.2.8. Determination of Polymeric Structural Variations Using Fourier Transmission Infrared Spectroscopy (FTIR)

The molecular structures of the native polymers, the drug-polymer granulated blend, and the compressed drug-loaded MLMDT were analyzed using FTIR spectroscopy (Perkin Elmer Spectrum 100 Series, Beaconfield, UK) to elucidate any variations in vibrational frequencies and subsequent polymeric structure as a result of drug-polymer or even polymer-polymer interactions. Changes in the polymeric backbone may affect the physicochemical and physicomechanical properties of the polymer and therefore such changes need to be determined. Analyses were performed in triplicate. 

#### 2.2.9. Surface Morphological Analysis

The surface morphology of the MLMDT was observed using Scanning Electron Microscopy (SEM). Samples of the MLMDT layers were sectioned using a surgical scalpel (to minimize interference) and mounted onto stubs and sputter coated with gold in a vacuum evaporator (Module Sputter Coater, SPI Supplies, PA, USA) and then photographed using a bench-top scanning electron microscope (Phenom Fei Company, OR, USA). 

#### 2.2.10. Atomistic Molecular Structural Mechanics Simulations

Molecular mechanics computations in vacuum, which included the model building of the energy-minimized structures of multipolymer complexes, were performed using the HyperChem 8.0.8 Molecular Modeling System (Hypercube Inc., Gainesville, FL, USA) and ChemBio3D Ultra 11.0 (CambridgeSoft Corporation, Cambridge, UK) [43]. The structures of ethylcellulose (EC), hydroxyethylcellulose (HEC), pectin (PEC), and Avicel (cellulose, AVC) (4 saccharide units each) were built from standard bond lengths and angles using the sugar builder module on HyperChem 8.0.8. The generation of the overall steric energy associated with the energy-minimized structures was initially executed via energy minimization using MM+ force field and the resulting structures were again energy minimized using the AMBER 3 force field. A complex of one molecule with another was assembled by disposing them in a parallel way, and the same procedure of energy minimization was repeated to generate the final models: EC-HEC and PEC-AVC. Full geometry optimizations were carried out in vacuum employing the Polak-Ribiere conjugate gradient method until an RMS gradient of 0.001 kcal/mol was reached. Force field options in the AMBER (with all H-atoms explicitly included) and MM+ (extended to incorporate nonbonded limits and restraints) methods were the HyperChem 8.0.8 defaults. For computations of energy attributes, the force fields were utilized with a distance-dependent dielectric constant scaled by a factor of 1. The 1–4 scale factors are the following: electrostatic 0.5 and Van der Waals 0.5 [43].

## 3. Results and Discussion

### 3.1. In-Process Validation Tests

The MLMDTs were uniform in mass, each having an average weight of 99.6 ± 0.4 mg (Table 4). The thickness ranged from 1.78 ± 0.02 to 5.18 ± 0.03 mm while friability was at an average of 0.5 ± 0.03% (i.e., within the set limit 1%) (Table 4), demonstrating desirable matrix compressibility.

### 3.2. Employment of an ANN Approach for Formulation Optimization

Results obtained from the model including the average of the MSE values for all the training runs and the best network run out of 10,000 epochs are highlighted in Tables 5(a) and 5(b), respectively. The correlation coefficient of *R* ^2^ = 0.98 (Table 5(c)) determined from the comparison between the desired output and actual output of *k*_3_ suggests that the employed training model was extremely efficient. 

**Figure 4:**
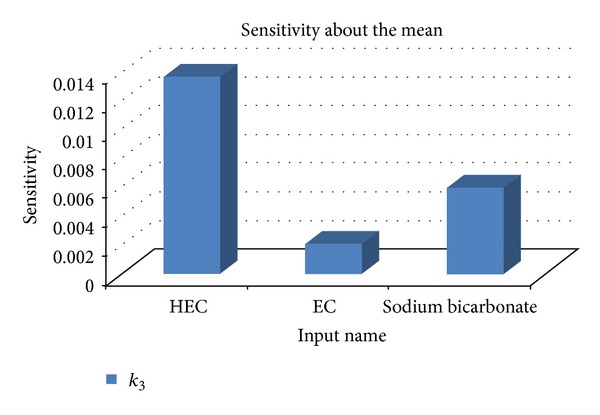
A typical bar chart portraying the ANN-derived sensitivity of HEC, EC, and sodium bicarbonate on *k*_3_.

**Figure 5:**
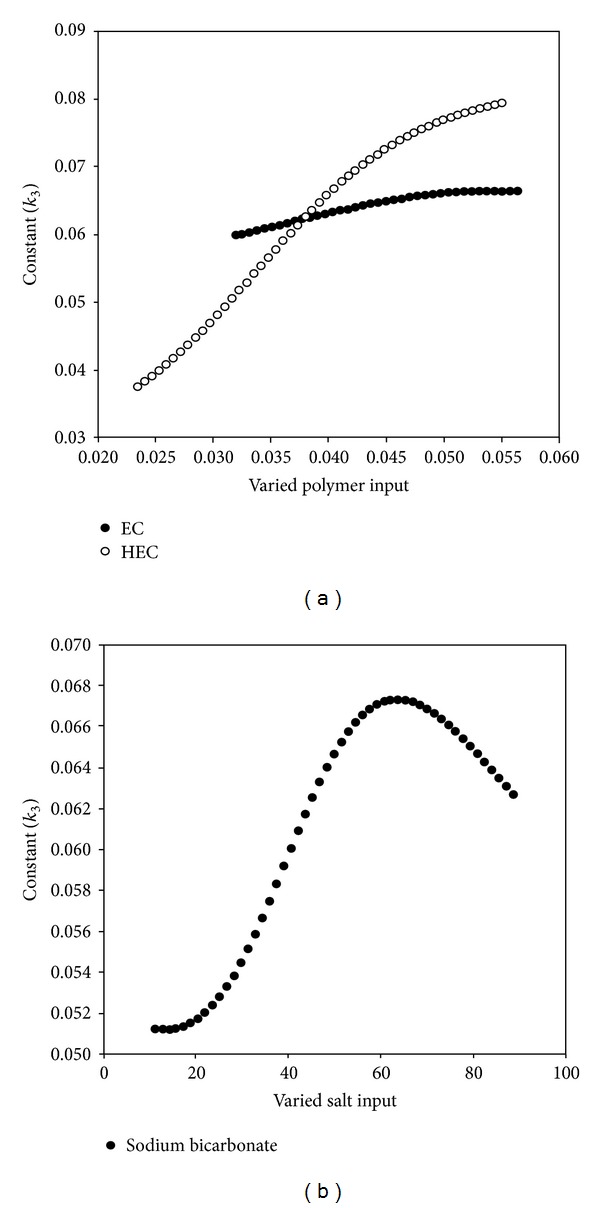
(a) Profiles showing the effect of EC and HEC on *k*_3_; (b) profile showing the influence of sodium bicarbonate on *k*_3_.

**Figure 6:**
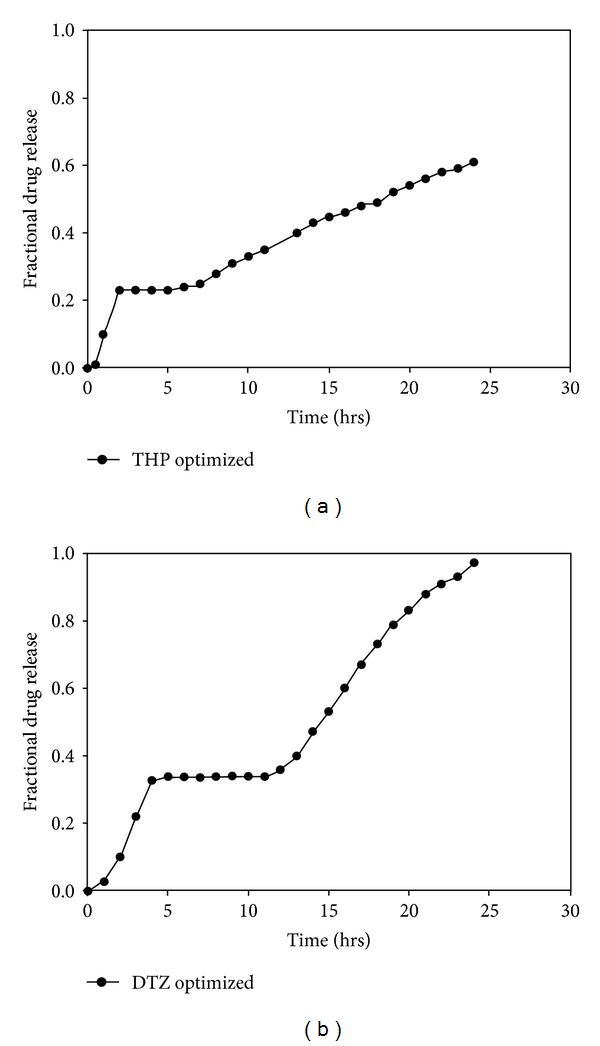
Drug release profiles of optimized (a) THP and (b) DTZ-loaded MLMDT formulations.

Results from sensitivity testing suggested that the quantity of HEC in the polymer disk had the most influence on the shape of the drug release profile (Figure 4). This was substantiated in the profile shown in Figure 5(a) which showed a linear curve for HEC, suggesting that the concentration of HEC greatly affected the drug release. Increasing the concentration of EC had a minor effect on the drug release. The quantity of sodium bicarbonate present in the DTZ formulation also affected the drug release profile as illustrated in Figure 5(b). There was a linear increase in the sensitivity of the MLMDT as the concentration of sodium bicarbonate increased up to 50 mg. However, at concentrations greater than 50 mg per layer, sodium bicarbonate had no longer a beneficial matrix-stiffening effect on the formulation. This suggested that 50 mg per layer was the optimum concentration. The compositions of the optimized formulation as determined by the ANN approach are depicted in Table 5(d), while drug release profiles are depicted in Figure 6. All further experiments were performed on the optimized THP-loaded and DTZ-loaded formulations.

### 3.3. In Vitro Drug Release Studies

Based on previous preliminary studies, drug release profiles were separated into four phases, namely, the initial phase, the first pulse of drug release, the lag phase, and second phase of drug release (Figure 2). Consequently, the rate release constant (*k*) for each phase was calculated based on the power law expression (4) describing drug release from simple swellable matrix systems. Consider

(4) $$\begin{array}{c} \frac{M_{t}}{M_{\infty }}=k_{1}t^{n}, \end{array}$$

where *M*_*t*_/*M*_*∞*_ is the fraction of drug released at time *t*, *k* is the release rate constant, and *n* is the release rate exponent. 

The rate constant, pertaining to the initial lag phase, first phase of drug release, “turn-off” phase, and last phase of drug release were termed *k*_1_, *k*_2_, *k*_3_, and *k*_4_, respectively. Ideally *k*_3_ should be 0.  Table 6 highlights the rate constants of the 21 chosen formulations. 

**Figure 7:**
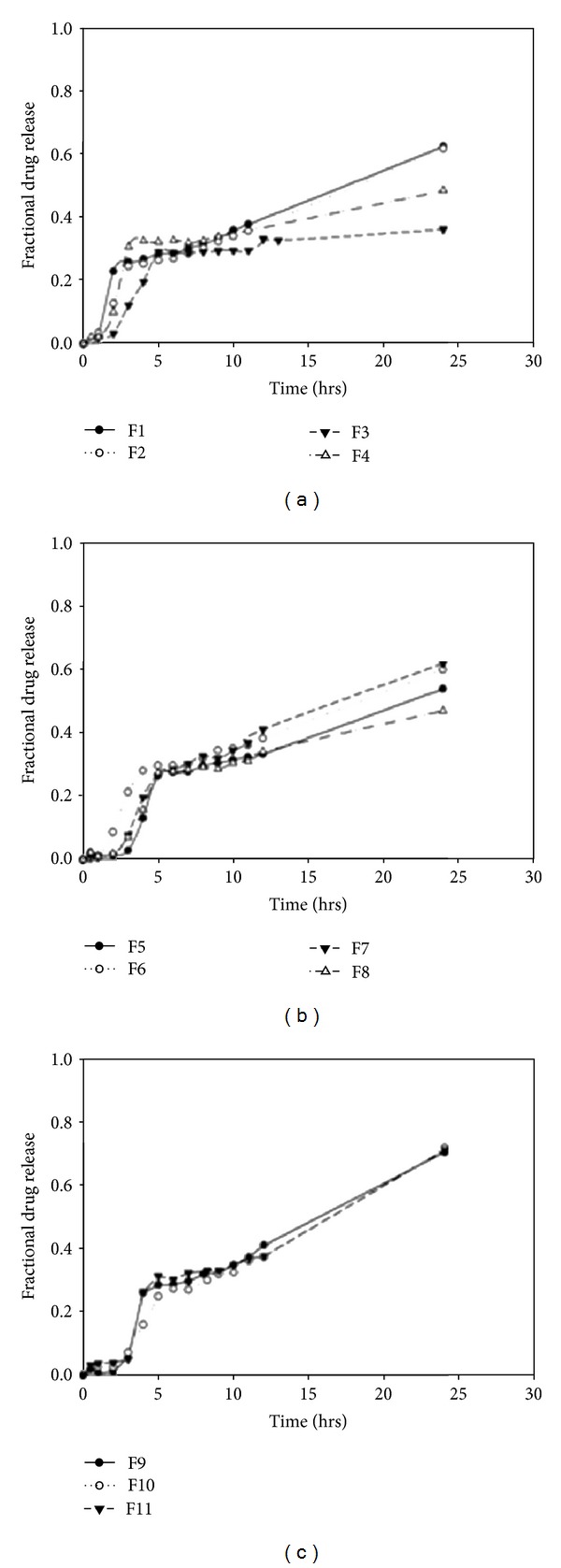
Drug release profiles of THP-loaded MLMDTs in pH 1.2 (2 hrs) and pH 6.8 (3 hours onwards).

**Figure 8:**
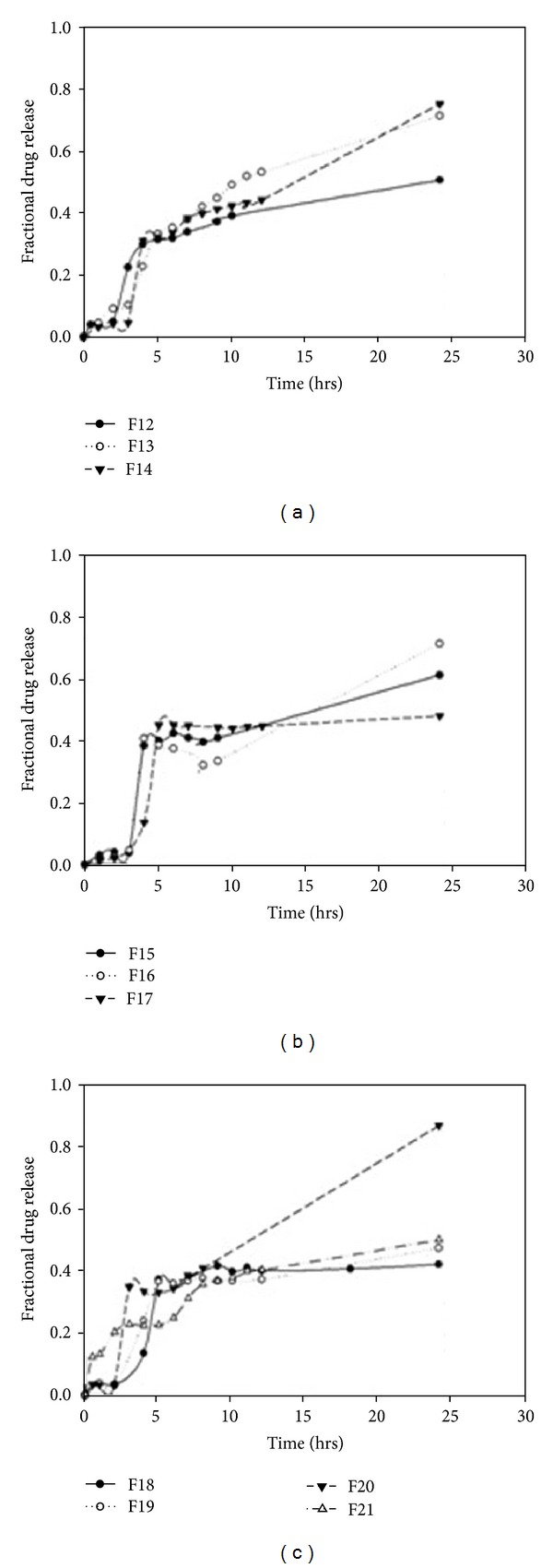
Drug release profiles of DTZ-loaded MLMDTs in pH 1.2.

Drug release profiles indicate that the formulations displayed an initial lag phase (Figures 7 and 8) due to the barrier layers surrounding the disks. Thereafter there was a rapid increase in drug release which was due to erosion of the PBL and the subsequent exposure of the lactose disk to the media. After complete release of drug from the lactose disk, drug release from all the formulations slowed down and then gradually increased. The release of drug from the polymer disk was controlled by the barrier layers and the type of polymer in the polymer disk. Due to the high hydrophobic nature of EC, formulations containing pure EC (F numbers 3, 4, 5, 6, 8 and 12) displayed reduced drug release (<50%) over the 24-hour period (Figures 7(a), 7(b), and 8(a)). Formulations for which hydrophilic polymers were employed (F numbers 1, 2, 7 and 9) demonstrated higher rates of drug release. However, instead of a lag phase, biphasic release was observed (Figures 7(a) and 7(c)). Utilizing both hydrophobic and hydrophilic polymers (F numbers 10, 11, 13, 14, 15, 16, 17, 18, 19, 20 and 21) provided a moderate rate of drug release that was fairly controlled.

#### 3.3.1. The Effect of Sodium Bicarbonate on Drug Release from DTZ-Loaded MLMDTs

DTZ is known for its high water solubility (>50% *w*/*w* at 25°C) making controlled release a challenge. To counteract this challenge, an electrolyte sodium bicarbonate was incorporated into the formulation to control the rate of release of DTZ as demonstrated by the mechanism proposed by Pillay and Fassihi, 1999 [44]. The location and quantity of sodium bicarbonate in the MLMDT significantly affected the rate of drug release as demonstrated by F numbers 14–20 (Figures 8(a), 8(b), and 8(c)). Higher concentrations of sodium bicarbonate (F numbers 17 and 18) reduced drug release to a level sufficient for incomplete release after the 24-hour period. Formulations that served as controls, that is, had no sodium bicarbonate (F numbers 12 and 21), displayed biphasic release instead of the desired lag phase.

### 3.4. Swelling and Erosion Studies

**Figure 9:**
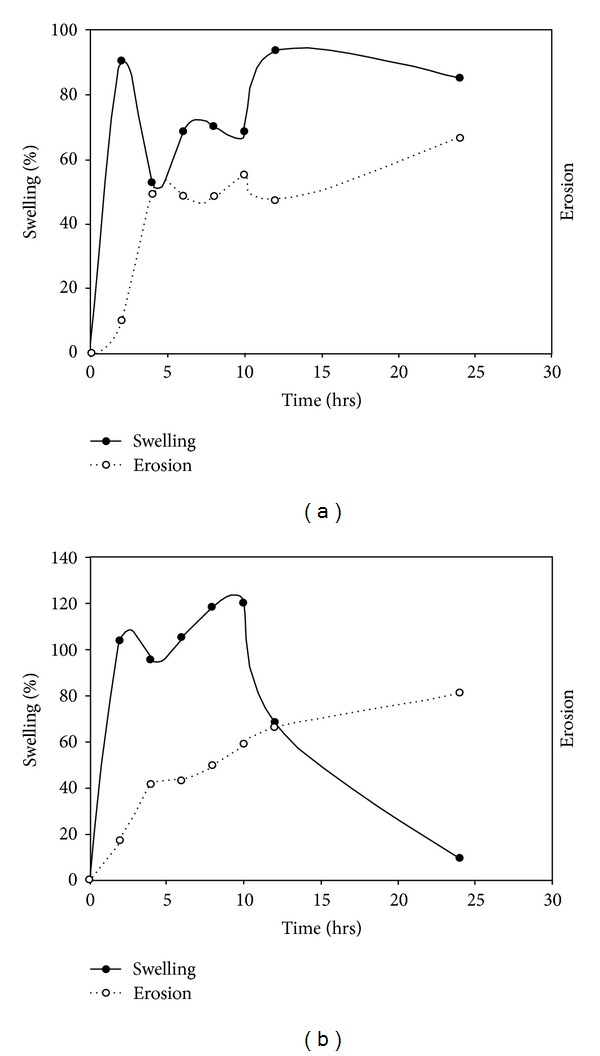
Correlation of swelling and erosion profiles of (a) THP-loaded and (b) DTZ-loaded MLMDTs.

**Figure 10:**
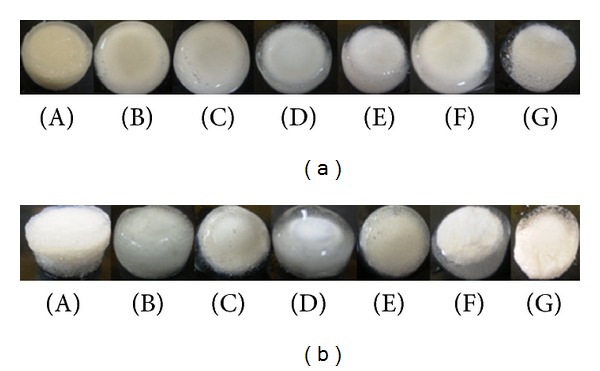
Digital images depicting (a) swollen THP-loaded MLMDTs and (b) swollen DTZ-loaded MLMDTs at (A) 2, (B) 4, (C) 6, (D) 8, (E) 10, (F) 12, and (G) 24 hours, respectively.

In order to obtain further evidence for the observed drug release kinetics, swelling and erosional studies were performed on the optimized formulations.  Figure 9 shows the relationship between swelling and erosion on (a) THP and (b) DTZ MLMDTs. The THP formulation swelled to 80% of the original size within the first two hours, with <10% of the MLMDT displaying erosion. It is this initial swelling that contributed to the lag phase seen in the drug release profiles (Figure 7(a)). After 4 hours though, there was a sharp decrease in the % of water intake (i.e., swelling). This may be ascribed to the erosion of the upper PBL layer. This was further substantiated by the digital images taken at *t* = 4 hours, which showed the absence of the upper layer and the lactose disk (Figure 10(a) (B)). After 6 hours, the HBL1 and HBL2 layers swelled steadily while the erosion rate remained reasonably constant. This accounted for the lag phase observed in the drug release profile. At 12 hours there was an increase in swelling, and at 24 hours a decrease in swelling was observed with an increase in erosion to approximately 60%. The MLMDT was relatively intact (Figure 10(a) (G)) after 24 hours, and this may explain the incomplete drug release that was obtained.

Swelling and erosion results from DTZ formulations display different results even though the same polymers were used. This was attributed to the inclusion of sodium bicarbonate in the DTZ formulation. Essentially, the addition of an alkaline salt to an acidic drug resulted in a buffering effect that inhibited drug release [45]. There was an increase in swelling within the first 2 hours contributing to the initial lag phase (Figure 8(a)). Subsequently, there was a drop in the swelling percentage similar to the THP-loaded MLMDTs due to the erosion of the upper PBL layer and the lactose disk. The DTZ formulation swelled >100% in the first 2 hours compared with the THP formulation. This suggested that the inclusion of sodium bicarbonate resulted in high matrix swelling. Thereafter there was a constant increase in the swelling behaviour as well as the erosion over the next 10 hours. This was confirmed by the digital images as shown in Figure 10(b). Despite the fact that there was an increase in swelling, minimal drug was released confirming that sodium bicarbonate does in fact inhibit drug release. After 12 hours, there was a sharp decrease in swelling which may be justified by the decreased rate in drug release (Figures 8(b) and 8(c)). After 24 hours, the amount of the device that remained intact was 20% *w*/*w* only indicating that most of the drug had been released thus correlating with the drug release profiles obtained.

### 3.5. Textural Analysis on the MLMDT

**Figure 11:**
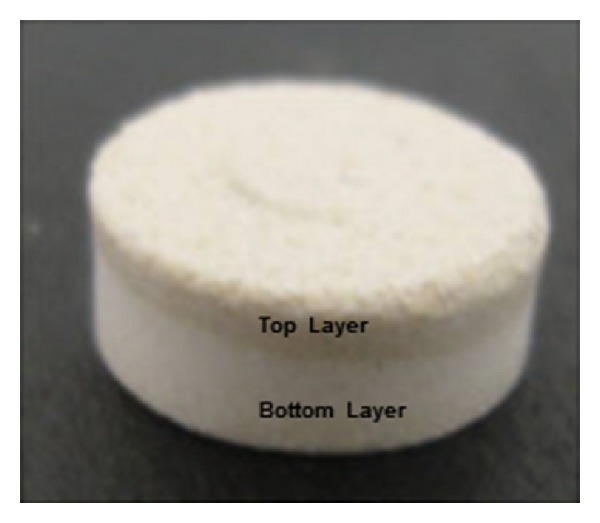
The MLMDT tablet showing the difference between the top and bottom layers of the device as based on the different polymers employed in the layers.

Textural analysis was performed to determine the indentation hardness which was converted to BHN. Since the MLMDTs comprised different polymers on the top and bottom layers (Figure 11), hardness was determined on both sides (top and bottom layers) of the MLMDT. According to the obtained results, the HBL2 layer proved to be considerably harder than the PBL layer (Table 7).

**Figure 12:**
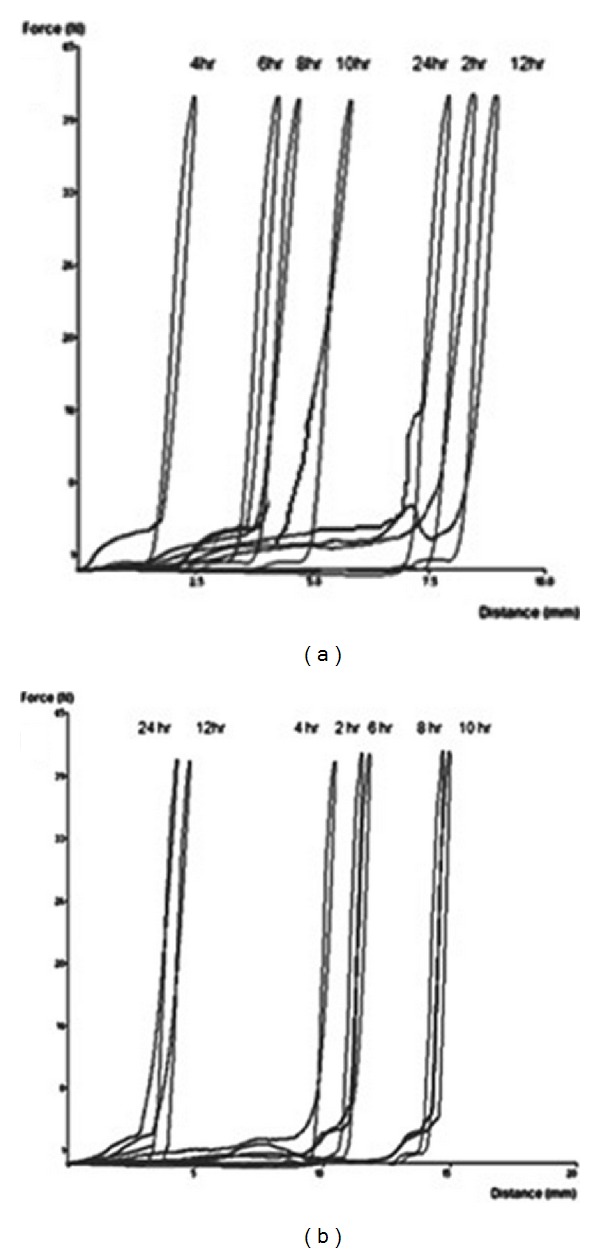
Typical force-displacement profiles for (a) THP-loaded MLMDTs in pH 1.2 and 6.8 and (b) DTZ-loaded MLMDTs in pH 1.2.

Textural analysis was also employed to measure alterations to swelling behavior. Force-displacement profiles for optimized DTZ- and THP-loaded formulations were obtained using the texture exponent software V2 (Figures 12(a) and 12(b)).  Figure 12(a) illustrates the force-displacement placement profiles for THP-loaded formulations exposed to pH 1.2 and 6.8, while Figure 12(b) displays the profile for DTZ-loaded formulations exposed to pH 1.2. The upward curving of the profile suggested the force needed to penetrate the swollen matrix, with a smaller force needed to penetrate the gel layer which increased once the probe penetrated the dry core. The rapid decline in the curves indicated the retraction of the probe from the swollen matrix (Figures 12(a) and 12(b)).

THP-loaded formulations swelled significantly within the first 2 hours followed by a decline in size. This corresponds to the erosion of the PBL layer. The MLMDT swelled progressively with the final measurement at 24 hours demonstrating a decline in size. These sequential events coincide with the shape of the digital images obtained in the % of water uptake (swelling) studies (Figure 12(a)). DTZ-loaded formulations displayed a considerable increase in swelling during the first 2 hours. This was followed by an increase in swelling up to 10 hours after which there was a decline in the swelling as erosion increased. The DTZ-loaded MLMDT swelled to a greater degree than the THP-loaded MLMDT. This may be ascribed to inclusion of sodium bicarbonate in the DTZ-loaded formulations and corresponds with the shape of the digital images obtained in Figure 12(b).

### 3.6. Determination of Polymeric Structural Variations Using Fourier Transmission Infrared Spectroscopy (FTIR)

**Figure 13:**
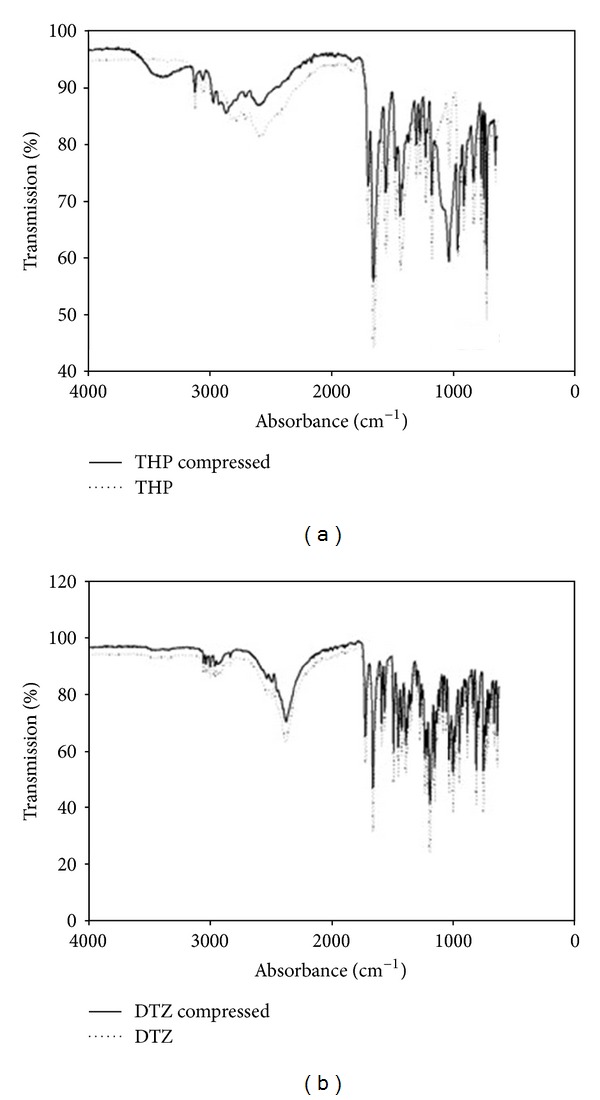
FTIR spectra depicting (a) pure THP and compressed THP MLMDT and (b) pure DTZ and compressed DTZ-loaded MLMDT.

FTIR spectroscopy was carried out on the native polymer and drug as well as the granulated (not shown) and compressed forms of the formulation. The spectra were then evaluated to determine if any changes in any of the structures occurred. FTIR spectra of both the compressed THP formulation (Figure 13(a)) and the compressed DTZ formulation (Figure 13(b)) show no change in the structure of the compressed final tablet in comparison with that of the native polymer and drug. Both figures illustrated characteristic cellulose bands occurring at 3476 cm^−1^, 2934 cm^−1^, and 1054 cm^−1^ typical of EC, HPMC, and HEC. Figure 13(a) shows the native THP peaks which were observed between 2500 cm^−1^ and 3100 cm^−1^, between 1500 cm^−1^ and 1700 cm^−1^, and at 700 cm^−1^, and they were unchanged in the compressed formulation. Similarly Figure 15(b) highlights the characteristic DTZ bands observed between 1600 cm^−1^ and 1800 cm^−1^, indicative of the stretching of the carbonyl bond in the amide group, which was present in the compressed tablet. Thus, the observed spectrum could be regarded as a simple superimposition between the native polymers and drug, suggesting that no interaction occurred between the polymer and the drug.

### 3.7. Surface Morphology Analysis on the Compressed MLMDT

**Figure 14:**
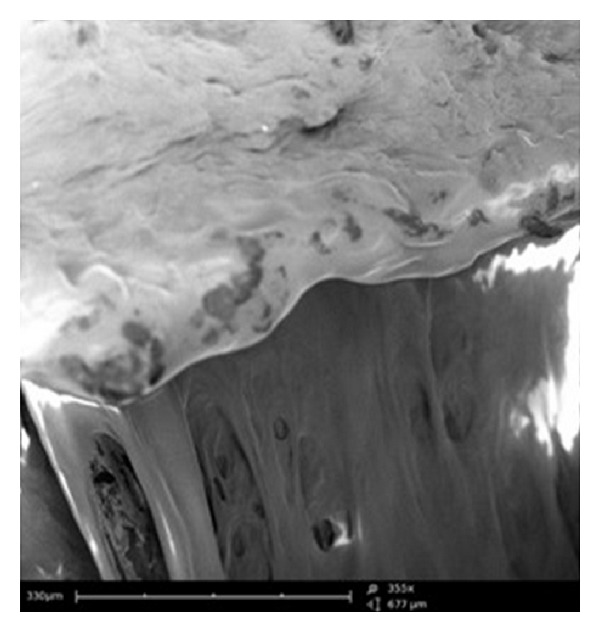
SEM image illustrating morphology of the pectin/Avicel (PBL) and HPMC (HBL1) layers (magnification 355x).

SEM was conducted on the compressed MLMDT to observe the surface morphology of the different layers. As highlighted in Figure 14, there was a distinct difference in the surface structure of the PBL and HBL1 layers. This variation in structure accounted for the different rates of erosion and swelling and consequently the drug release kinetics.

### 3.8. Molecular Mechanics Assisted Model Building and Energy Refinements

#### 3.8.1. Molecular Mechanics Energy Relationship Analysis

The analytico-mathematical illustration of the potential energy values, in the form of molecular mechanics energy relationship (MMER) analysis, was employed to gain mechanistic information involved in the bonding and nonbonding contributors. Blended-polysaccharide morphologies and interactions were explained with reference to valence terms, coulombic terms, and London dispersion forces. The MMER model for the various steric energy factors inherent to the molecular complexes can be written as (5)–(7). Consider the following:

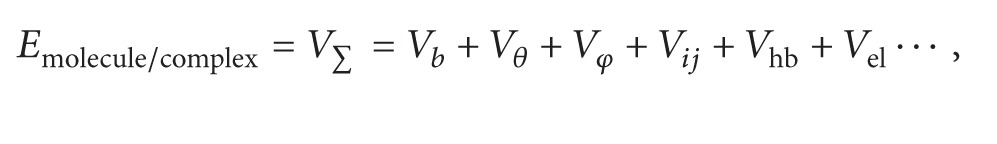
(5)

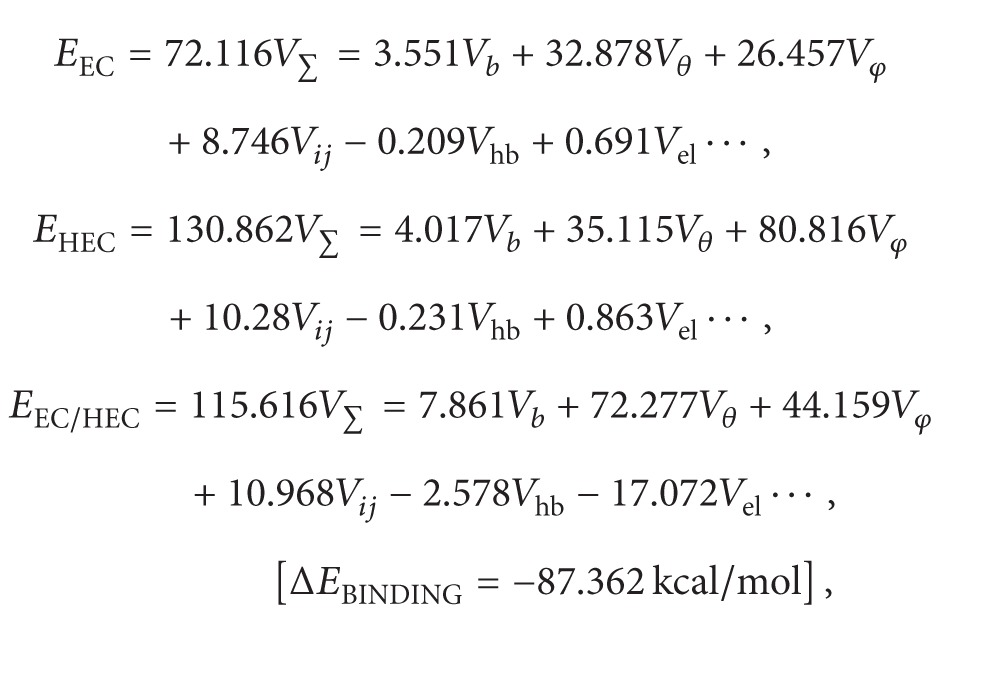
(6)

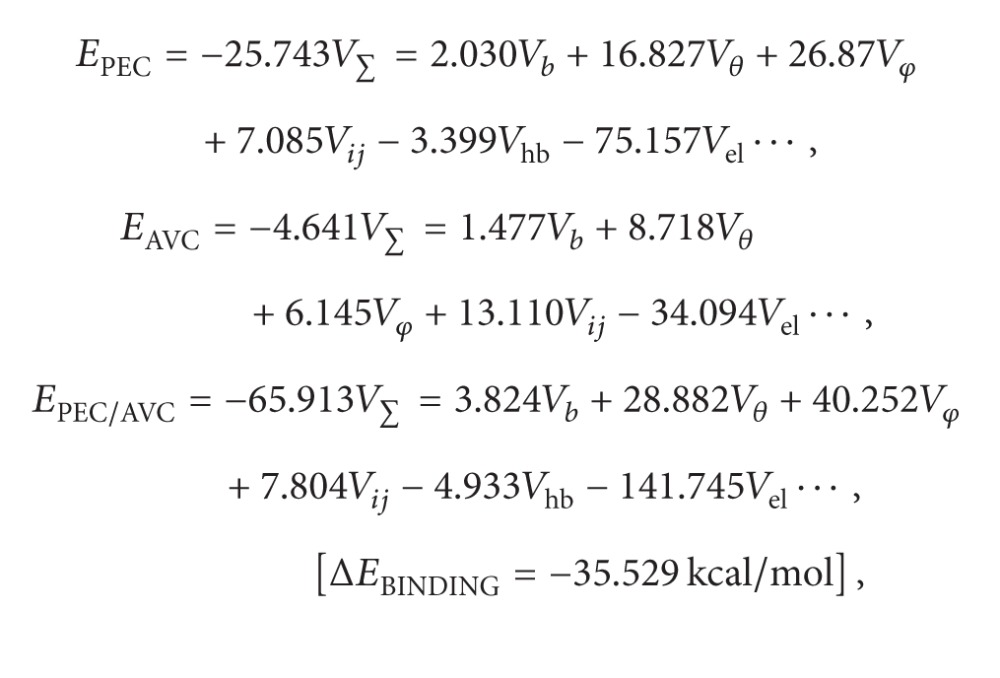
(7)
where *V*_∑ _ = total steric energy for an optimized structure, *V*_*b*_ = bond stretching contributions, *V*_*θ*_ = bond angle contributions, *V*_*φ*_ = torsional contributions, *V*_*ij*_ = van der Waals interactions, *V*_hb_ = hydrogen-bond energy function, and *V*_el_ = electrostatic energy.

#### 3.8.2. Geometrical Optimizations for Composite Polysaccharide Morphologies

**Figure 15:**
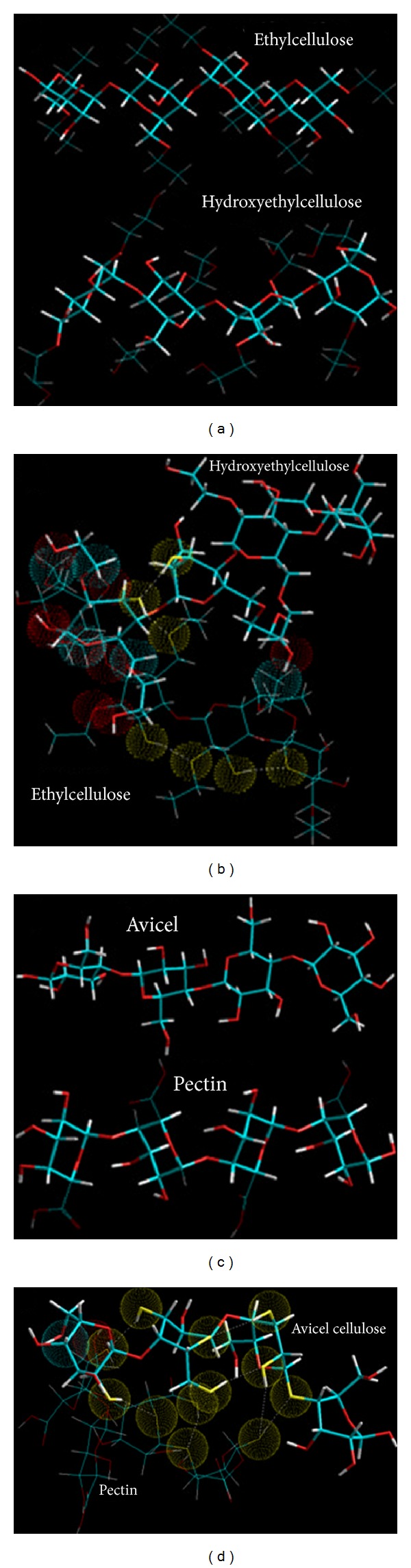
Energy minimized geometrically constrained models of the following. (a) Ethylcellulose and hydroxyethylcellulose before complexation; (b) EC-HEC complex derived from MM computations. The atoms in close interaction proximity are emphasized by space filling model (dots) where the yellow dots depict atoms involved in H-bonding. Color codes for elements are carbon (cyan), nitrogen (blue), oxygen (red), and hydrogen (white). (c) Avicel (cellulose) and pectin before complexation; (d) PEC-AVC complex derived from MM computations. The atoms in close interaction proximity are emphasized by space filling model (dots) where the yellow dots depict atoms involved in H-bonding. Color codes for elements are carbon (cyan), nitrogen (blue), oxygen (red), and hydrogen (white).

The molecular geometry minimized structures of the EC-HEC and PEC-AVC after static lattice atomistic iterations were modeled as depicted in Figures 15(a)–15(d), and the respective steric energy values to which they will be responsive are listed in (7). Molecular modeling studies may assist in determination of the specific interactions among component polymer segments and may further provide estimation towards the compatibility of the blending polymers. Our theoretical foundation for this method relies upon the energetic factors influenced by the thermodynamics of the interactions acting locally among the segments of the polymer chains involved in modeling computations [46].

The total steric energy value for the EC-HEC complex (representing base disc) is stabilized by a binding energy of ~87 kcal/mol (Δ*E*_BINDING_ = −87.362 kcal/mol; (6)). On a theoretical basis, the mandatory condition determining the compatibility and miscibility of a blend of two polymers is related to the negative free energy of mixing, that is, –ve Δ*E* [46]. This energy minimization in case of EC-HEC was caused due to the torsional contributions involved among the interacting monosaccharide residues producing rotational strain due to steric interactions. These torsional strains were however dismissed by the introduction of bond length and bond angle alterations with respect to the systems' degrees of freedom. As obvious from comparison between Figures 15(a) and 15(b), the lowering of the energetic obstructions leads to a substantial change from the initial starting geometry. The pendent groups (ethyl and hydroxylethyl) moved to their “nearest minimum downhill” from the starting point during minimization and hence causing the molecules to pass through unfavorable regions. The steric modulations further assist the pendent groups to overcome the torsional barriers presenting a larger accessible potential energy surface [47]. This torsional energy stabilization (Δ*E*_*V*_*φ*__ ~ 62 kcal/mol) contributed mainly to the finally stabilized geometrical configurations. These energy optimizations are also supported by high magnitude van der Waals interactions (Δ*E*_*V*_el__ ~ 20 kcal/mol) between the two 4-saccharide units sugar molecules. Furthermore, regarding the spatial preference of EC with HEC, as depicted by the dots rendering in Figure 15(b), a closer look at the EC-HEC molecular structure revealed the close proximity of both the polymeric molecules in form of H-bonding and those closely sharing the van der Waals space.

These underlying weak chemical interactions may not cause a structural change in the polymers but may initiate aggregation of the aliphatic chains, as both the polymers here contain aliphatic side chains, hence creating relocalized areas with density and refractive index different from the initial values. Additionally, the hydroxyl group induced inter- and intramolecular hydrogen bonding in EC-HEC (*≈*10 times more than the individual polymers) (Figure 15(b)) which may influence the hydration process of the EC polymer matrix causing a “notable increase in the amount of drug released over a given period” as explained earlier in the paper. This release pattern may be attributed to the modified matrix hydration process of EC (hydrogen bonding induced by the presence of the HEC) and continued retarded release of drug due to the entanglement of saccharide chains which in turn lead to a high BHN of layer 3 as shown in Table 7.

Similarly, the molecular mechanistically minimized energy value for PEC-AVC complex (representing layer 1) is stabilized by a binding energy of ~35 kcal/mol (Δ*E*_BINDING_ = −35.529 kcal/mol; (7)). Molecular modeling proved to be a powerful tool for studying the orientation of polysaccharides where the molecular models can be developed for the calculation of the energies involving chain-chain interactions which can be calculated as described by Pérez and coworkers, 1996 [47]. Additionally, in case of PEC-AVC, the pectin's interaction model with cellulose was generated by trying various helix translations along the axis and different mutual rotational orientations with the helices within van der Waals radius (Figures 15(c) and 15(d)). For efficient packing, low energy of stabilization was supported by the rotations caused by the coupled individual chains. These very rotations caused the formation of bonding interactions in the form of torsional energy minimization (~10 kcal/mol), the formation of H-bonds (~12 kcal/mol), and highly stabilized electrostatic interactions (~30 kcal/mol) leading to the formation of a rotational screw axis as depicted in Figures 15(c) and 15(d). The aforementioned interactions involving the nonbonded forces may cause the formation of induced dipoles within the complex. Additionally, the binding energy changes should be proportional to the polarizability of the substituents, which in turn may lead to the formation of a dense polymer network responsible for prolonged release of the bioactives (Figures 15(c) and 15(d)).

The higher energy of stabilization (Δ*E*_BINDING_) of EC-HEC as compared to PEC-AVC corroborated with the chronotherapeutic strategy explained in this research. PEC-AVC being a less stabilized molecular complex is anticipated to erode faster than EC-HEC leading to the release of drug from layer 1 earlier than that from base disc. Hence, the present modeling and computation method involving four polysaccharides provided a justification of using a definite combination of polymers to meet the requirements of a “drug delivery system with desired release profile (DDSDRP).” This MLMDT and subsequent modeling system may act as a template for the future applications employing various drug and polymer combination strategies involving chronotherapeutic and DDSDRP requirements.

## 4. Conclusions

The MLMDT was successfully developed and characterized. The system comprised two drug-loaded disks enveloped by barrier layers. Friability, hardness, and uniformity of mass were all within the specified limits depicting desirable manufacturing settings. The MLMDT provided two pulses of drug release, separated by a lag phase. The lag phase was dependent on the polymers utilized in the polymer disk, with the concentrations of HEC and sodium bicarbonate being the most important factors in producing the lag phase. The experimental results were well corroborated by the molecular mechanics computations. Overall, the MLMDT showed much promise as a chronotherapeutic drug delivery system.

## Figures and Tables

**Table 1:** Mass constituents comprising the MLMDT for both DTZ and THP formulations.

	DTZ MLMDT	THP MLMDT
Pectin barrier layer (PBL)	150 mg	150 mg
HPMC middle barrier layer 1 (HBL1)	150 mg^1^	100 mg
HPMC outer barrier layer 2 (HBL2)	250 mg^1^	200 mg
Lactose disk	160 mg	160 mg
Polymer disk (EC/HEC)	160 mg	160 mg

Total weight of tablet	870 mg	770 mg

^1^The DTZ MLMDT includes an additional 100 mg of sodium bicarbonate in the HPMC layers.

**Table:** (a)

Formulation	HEC (mg)	EC (mg)	HPMC (mg)	Granulating agent		Bulking agent
				Surelease	Sureteric	
1	—	—	70	—	—	—
2	70	—	—	*✓*	*✓*	—
3	—	70	—	—	*✓*	—
4	—	70	—	*✓*	—	—
5	—	50	—	*✓*	—	*✓*
6	—	35	—	*✓*	—	*✓*
7	70	—	—	*✓*	—	—
8	—	23	—	*✓*	—	*✓*
9	—	—	70	*✓*	—	—
10	35	35	—	*✓*	—	—
11	14	56	—	*✓*	—	—

**Table:** (b)

Formulation	HEC (mg)	EC (mg)	Surelease	Sodium bicarbonate			
				Quantity	Location		
					Polymer disk (mg)	HBL1^1^	HBL2^2^
12	—	84	*✓*	—	—	—	—
13	21	63	*✓*	—	—	—	—
14	21	63	*✓*	100	—	*✓*	*✓*
15	21	63	*✓*	50	*✓*	—	—
16	21	63	*✓*	150	*✓*	*✓*	*✓*
17	21	63	*✓*	200	—	*✓*	*✓*
18	21	63	*✓*	300	*✓*	*✓*	*✓*
19	21	63	*✓*	100	*✓*	—	—
20	63	21	*✓*	100	—	*✓*	*✓*
21	42	42	*✓*	—	—	—	—

^1^HBL1: HPMC middle barrier layer.

^2^HBL2: HPMC outer barrier layer.

Surelease: ethylcellulose.

Sureteric: polyvinyl acetate phthalate.

**Table 3:** Textural settings employed for the determination of deformation energy and matrix hardness.

Parameter	Settings	
	Deformation energy	Matrix hardness
Pretest speed	1 mm/sec	1 mm/sec
Test speed	0.5 mm/sec	0.5 mm/sec
Posttest speed	10 mm/sec	1 mm/sec
Compression force	40 N	40 N
Trigger type	Auto	Auto
Trigger force	0.05 N	0.01 N
Load cell	5 kg	5 kg

**Table 4:** Friability, thickness, and mass uniformity for the DTZ and THP-loaded formulations (*N* = 10).

Formulation	Friability (%)	Thickness (mm)	Uniformity of mass^1^ (%)
THP MLMDT	0.3	4.82 ± 0.03	99.6 ± 0.4
THP lactose disk	0.9	1.78 ± 0.02	99.4 ± 0.6
THP polymer disk	0.5	2.08 ± 0.02	99.6 ± 0.4
DTZ MLMDT	0.3	5.18 ± 0.03	99.7 ± 0.3
DTZ lactose disk	0.8	1.78 ± 0.02	99.9 ± 0.1
DTZ polymer disk	0.4	2.14 ± 0.04	99.5 ± 0.5

^1^Expressed as a percentage of the theoretical weight.

**Table:** (a)

All runs	Training minimum	Training standard deviation
Average of minimum MSEs	0.016	0.0059
Average of final MSEs	0.016	0.0059

**Table:** (b)

Best network	Training
Run number	2
Epoch number	1000
Minimum MSE	0.0098
Final MSE	0.098

**Table:** (c)

Performance	*k*_3_
MSE	4.51*E* − 06
NMSE	0.056
MAE	0.0015
Min abs error	0.00011
Max abs error	0.0053
Correlation coefficient *R* ^2^	0.98

**Table:** (d)

Sodium bicarbonate						
Drug	Polymer	Polymer	Ratio	Quantity	HBL1^1^	HBL2^2^
THP	HEC	EC	1 : 3	—	—	—
DTZ	HEC	EC	1 : 1	100	*✓*	*✓*

^1^HBL1: HPMC middle barrier layer.

^2^HBL2: HPMC outer barrier layer.

**Table 6:** The rate constants of the various formulations.

	*k*_1_	*k*_2_	*k*_3_	*k*_4_
1	0.031	0.089	0.042	0.026
2	0.034	0.069	0.040	0.026
3	0.018	0.049	0.034	0.015
4	0.038	0.092	0.049	0.020
5	0.018	0.040	0.039	0.028
6	0.056	0.071	0.048	0.033
7	0.018	0.047	0.034	0.026
8	0.022	0.045	0.031	0.020
9	0.018	0.053	0.036	0.029
10	0.021	0.044	0.034	0.030
12	0.048	0.075	0.049	0.021
13	0.042	0.062	0.051	0.030
14	0.017	0.12	0.063	0.029
15	0.021	0.095	0.060	0.025
17	0.013	0.062	0.051	0.027
18	0.027	0.055	0.042	0.020
19	0.010	0.067	0.045	0.022
20	0.038	0.010	0.057	0.036
21	0.019	0.090	0.042	0.021

**Table 7:** Brinell Hardness Numbers for the MLMDTs.

Formulation	BHN (N/mm^2^)
THP MLMDT layer one (PBL)	5.35
THP MLMDT layer three (HBL2)	6.49
DTZ MLMDT layer one (PBL)	5.27
DTZ MLMDT layer three (HBL2)	6.60
